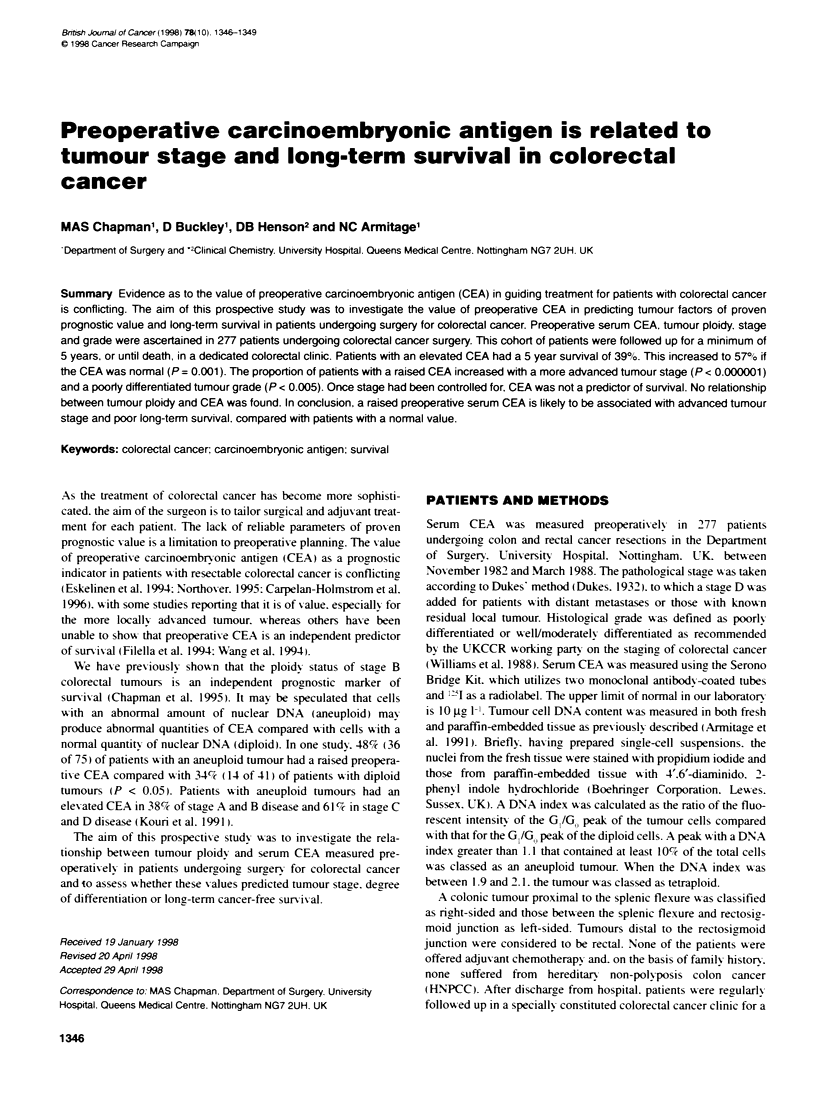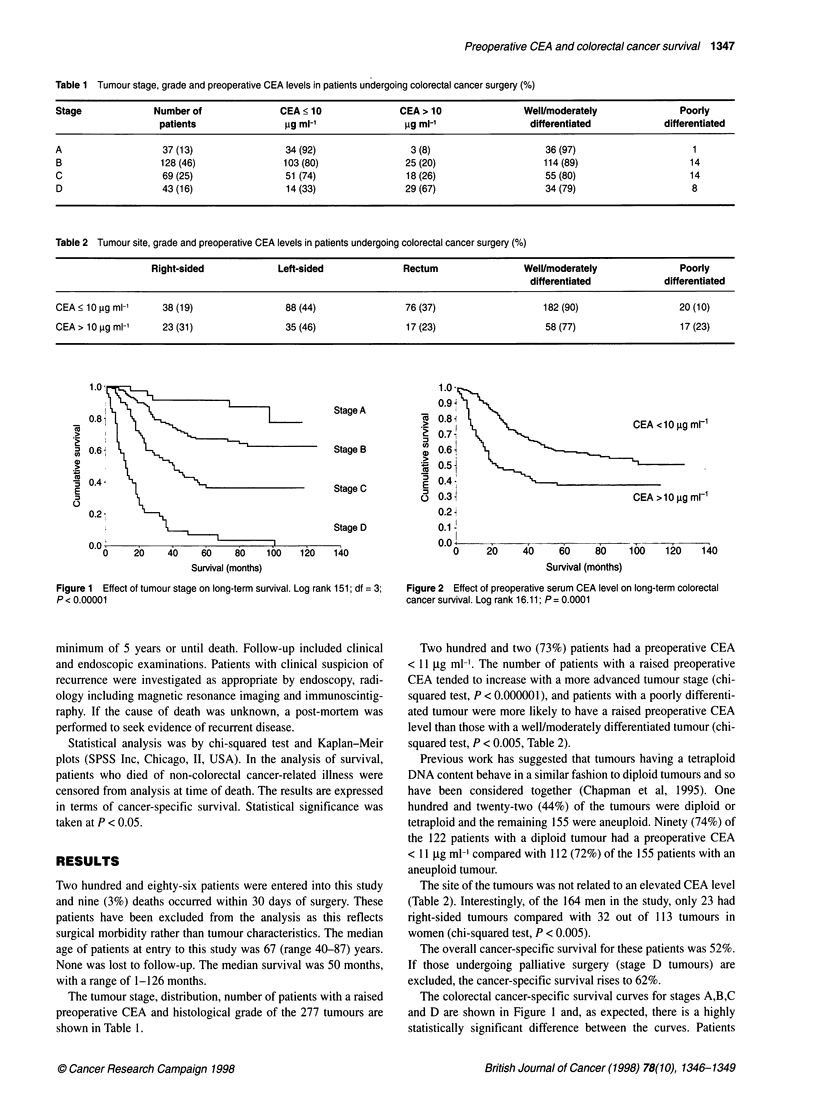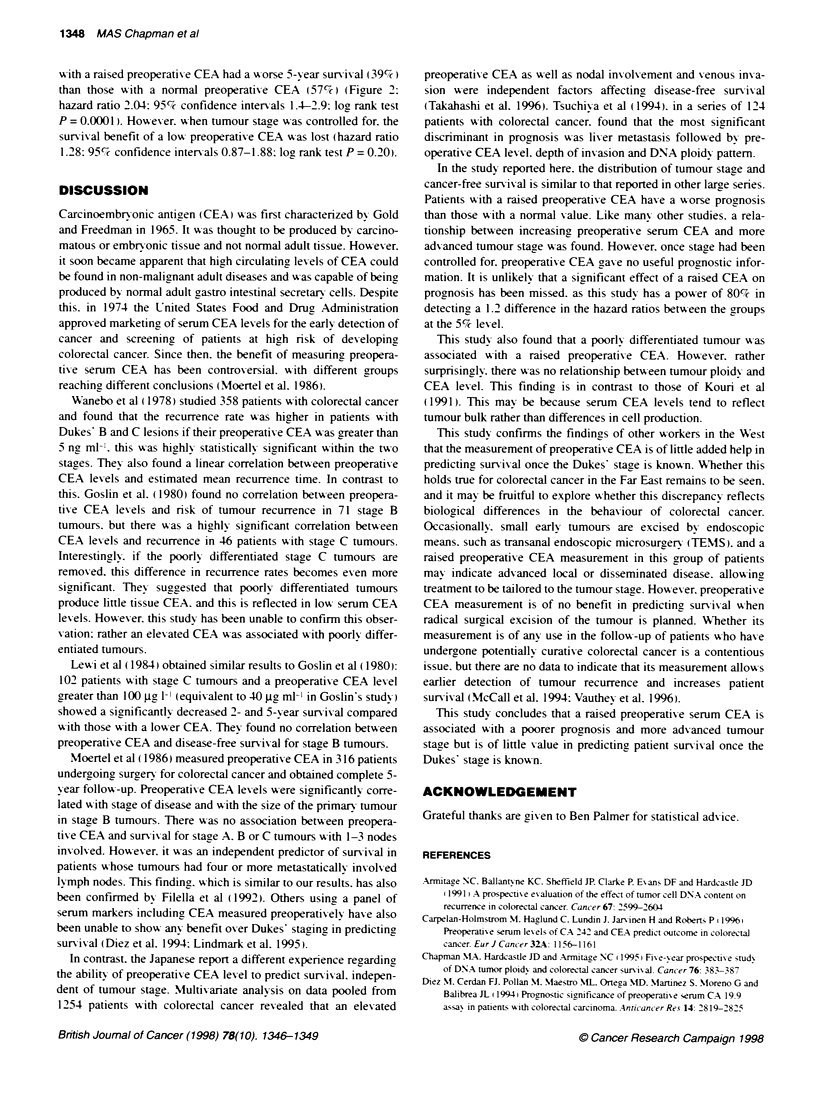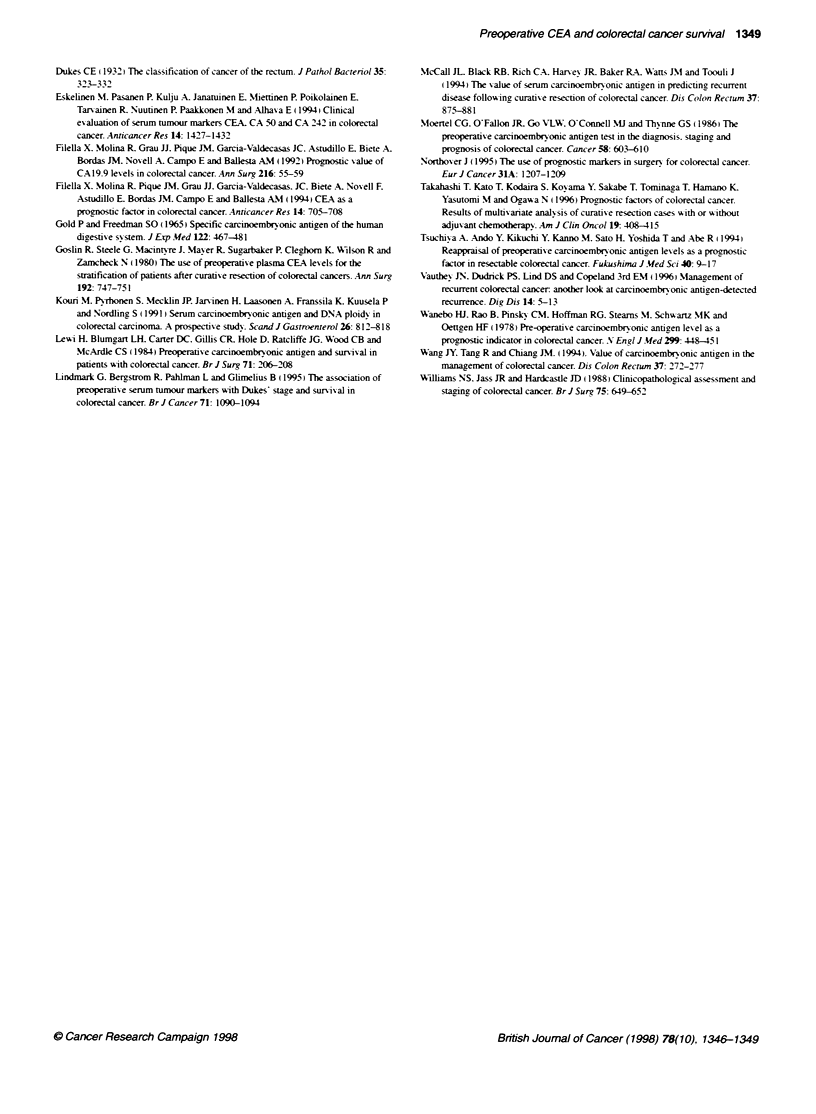# Preoperative carcinoembryonic antigen is related to tumour stage and long-term survival in colorectal cancer.

**DOI:** 10.1038/bjc.1998.682

**Published:** 1998-11

**Authors:** M. A. Chapman, D. Buckley, D. B. Henson, N. C. Armitage

**Affiliations:** Department of Surgery, University Hospital, Queens Medical Centre, Nottingham, UK.

## Abstract

Evidence as to the value of preoperative carcinoembryonic antigen (CEA) in guiding treatment for patients with colorectal cancer is conflicting. The aim of this prospective study was to investigate the value of preoperative CEA in predicting tumour factors of proven prognostic value and long-term survival in patients undergoing surgery for colorectal cancer. Preoperative serum CEA, tumour ploidy, stage and grade were ascertained in 277 patients undergoing colorectal cancer surgery. This cohort of patients were followed up for a minimum of 5 years, or until death, in a dedicated colorectal clinic. Patients with an elevated CEA had a 5 year survival of 39%. This increased to 57% if the CEA was normal (P=0.001). The proportion of patients with a raised CEA increased with a more advanced tumour stage (P < 0.000001) and a poorly differentiated tumour grade (P < 0.005). Once stage had been controlled for, CEA was not a predictor of survival. No relationship between tumour ploidy and CEA was found. In conclusion, a raised preoperative serum CEA is likely to be associated with advanced tumour stage and poor long-term survival, compared with patients with a normal value.


British Journal of Cancer (1998) 78(10), 1346–1349
© 1998 Cancer Research Campaign

### Preoperative carcinoembryonic antigen is related to tumour stage and long-term survival in colorectal cancer

**MAS Chapman[1], D Buckley[1], DB Henson[2] and NC Armitage[1]**

[1]Department of Surgery and [2]Clinical Chemistry, University Hospital, Queens Medical Centre, Nottingham NG7 2UH, UK

**Summary** Evidence as to the value of preoperative carcinoembryonic antigen (CEA) in guiding treatment for patients with colorectal cancer is conflicting. The aim of this prospective study was to investigate the value of preoperative CEA in predicting tumour factors of proven prognostic value and long-term survival in patients undergoing surgery for colorectal cancer. Preoperative serum CEA, tumour ploidy, stage and grade were ascertained in 277 patients undergoing colorectal cancer surgery. This cohort of patients were followed up for a minimum of 5 years, or until death, in a dedicated colorectal clinic. Patients with an elevated CEA had a 5 year survival of 39%. This increased to 57% if the CEA was normal ($P = 0.001$). The proportion of patients with a raised CEA increased with a more advanced tumour stage ($P < 0.000001$) and a poorly differentiated tumour grade ($P < 0.005$). Once stage had been controlled for, CEA was not a predictor of survival. No relationship between tumour ploidy and CEA was found. In conclusion, a raised preoperative serum CEA is likely to be associated with advanced tumour stage and poor long-term survival, compared with patients with a normal value.

**Keywords:** colorectal cancer; carcinoembryonic antigen; survival

As the treatment of colorectal cancer has become more sophisticated, the aim of the surgeon is to tailor surgical and adjuvant treatment for each patient. The lack of reliable parameters of proven prognostic value is a limitation to preoperative planning. The value of preoperative carcinoembryonic antigen (CEA) as a prognostic indicator in patients with resectable colorectal cancer is conflicting (Eskelinen et al, 1994; Northover, 1995; Carpelan-Holmstrom et al, 1996), with some studies reporting that it is of value, especially for the more locally advanced tumour, whereas others have been unable to show that preoperative CEA is an independent predictor of survival (Filella et al, 1994; Wang et al, 1994).

We have previously shown that the ploidy status of stage B colorectal tumours is an independent prognostic marker of survival (Chapman et al, 1995). It may be speculated that cells with an abnormal amount of nuclear DNA (aneuploid) may produce abnormal quantities of CEA compared with cells with a normal quantity of nuclear DNA (diploid). In one study, 48% (36 of 75) of patients with an aneuploid tumour had a raised preoperative CEA compared with 34% (14 of 41) of patients with diploid tumours ($P < 0.05$). Patients with aneuploid tumours had an elevated CEA in 38% of stage A and B disease and 61% in stage C and D disease (Kouri et al, 1991).

The aim of this prospective study was to investigate the relationship between tumour ploidy and serum CEA measured preoperatively in patients undergoing surgery for colorectal cancer and to assess whether these values predicted tumour stage, degree of differentiation or long-term cancer-free survival.

*Received 19 January 1998*
*Revised 20 April 1998*
*Accepted 29 April 1998*

*Correspondence to:* MAS Chapman, Department of Surgery, University Hospital, Queens Medical Centre, Nottingham NG7 2UH, UK

### PATIENTS AND METHODS

Serum CEA was measured preoperatively in 277 patients undergoing colon and rectal cancer resections in the Department of Surgery, University Hospital, Nottingham, UK, between November 1982 and March 1988. The pathological stage was taken according to Dukes' method (Dukes, 1932), to which a stage D was added for patients with distant metastases or those with known residual local tumour. Histological grade was defined as poorly differentiated or well/moderately differentiated as recommended by the UKCCR working party on the staging of colorectal cancer (Williams et al, 1988). Serum CEA was measured using the Serono Bridge Kit, which utilizes two monoclonal antibody-coated tubes and $^{125}I$ as a radiolabel. The upper limit of normal in our laboratory is 10 $\mu g\ l^{-1}$. Tumour cell DNA content was measured in both fresh and paraffin-embedded tissue as previously described (Armitage et al, 1991). Briefly, having prepared single-cell suspensions, the nuclei from the fresh tissue were stained with propidium iodide and those from paraffin-embedded tissue with 4′,6′-diaminido, 2-phenyl indole hydrochloride (Boehringer Corporation, Lewes, Sussex, UK). A DNA index was calculated as the ratio of the fluorescent intensity of the $G_1/G_0$ peak of the tumour cells compared with that for the $G_1/G_0$ peak of the diploid cells. A peak with a DNA index greater than 1.1 that contained at least 10% of the total cells was classed as an aneuploid tumour. When the DNA index was between 1.9 and 2.1, the tumour was classed as tetraploid.

A colonic tumour proximal to the splenic flexure was classified as right-sided and those between the splenic flexure and rectosigmoid junction as left-sided. Tumours distal to the rectosigmoid junction were considered to be rectal. None of the patients were offered adjuvant chemotherapy and, on the basis of family history, none suffered from hereditary non-polyposis colon cancer (HNPCC). After discharge from hospital, patients were regularly followed up in a specially constituted colorectal cancer clinic for a

**Table 1** Tumour stage, grade and preoperative CEA levels in patients undergoing colorectal cancer surgery (%)

| Stage | Number of patients | CEA ≤ 10 μg ml$^{-1}$ | CEA > 10 μg ml$^{-1}$ | Well/moderately differentiated | Poorly differentiated |
|---|---|---|---|---|---|
| A | 37 (13) | 34 (92) | 3 (8) | 36 (97) | 1 |
| B | 128 (46) | 103 (80) | 25 (20) | 114 (89) | 14 |
| C | 69 (25) | 51 (74) | 18 (26) | 55 (80) | 14 |
| D | 43 (16) | 14 (33) | 29 (67) | 34 (79) | 8 |

**Table 2** Tumour site, grade and preoperative CEA levels in patients undergoing colorectal cancer surgery (%)

| | Right-sided | Left-sided | Rectum | Well/moderately differentiated | Poorly differentiated |
|---|---|---|---|---|---|
| CEA ≤ 10 μg ml$^{-1}$ | 38 (19) | 88 (44) | 76 (37) | 182 (90) | 20 (10) |
| CEA > 10 μg ml$^{-1}$ | 23 (31) | 35 (46) | 17 (23) | 58 (77) | 17 (23) |

**Figure 1** Effect of tumour stage on long-term survival. Log rank 151; df = 3; $P < 0.00001$

**Figure 2** Effect of preoperative serum CEA level on long-term colorectal cancer survival. Log rank 16.11; $P = 0.0001$

minimum of 5 years or until death. Follow-up included clinical and endoscopic examinations. Patients with clinical suspicion of recurrence were investigated as appropriate by endoscopy, radiology including magnetic resonance imaging and immunoscintigraphy. If the cause of death was unknown, a post-mortem was performed to seek evidence of recurrent disease.

Statistical analysis was by chi-squared test and Kaplan–Meir plots (SPSS Inc, Chicago, Il, USA). In the analysis of survival, patients who died of non-colorectal cancer-related illness were censored from analysis at time of death. The results are expressed in terms of cancer-specific survival. Statistical significance was taken at $P < 0.05$.

### RESULTS

Two hundred and eighty-six patients were entered into this study and nine (3%) deaths occurred within 30 days of surgery. These patients have been excluded from the analysis as this reflects surgical morbidity rather than tumour characteristics. The median age of patients at entry to this study was 67 (range 40–87) years. None was lost to follow-up. The median survival was 50 months, with a range of 1–126 months.

The tumour stage, distribution, number of patients with a raised preoperative CEA and histological grade of the 277 tumours are shown in Table 1.

Two hundred and two (73%) patients had a preoperative CEA < 11 μg ml$^{-1}$. The number of patients with a raised preoperative CEA tended to increase with a more advanced tumour stage (chi-squared test, $P < 0.000001$), and patients with a poorly differentiated tumour were more likely to have a raised preoperative CEA level than those with a well/moderately differentiated tumour (chi-squared test, $P < 0.005$, Table 2).

Previous work has suggested that tumours having a tetraploid DNA content behave in a similar fashion to diploid tumours and so have been considered together (Chapman et al, 1995). One hundred and twenty-two (44%) of the tumours were diploid or tetraploid and the remaining 155 were aneuploid. Ninety (74%) of the 122 patients with a diploid tumour had a preoperative CEA < 11 μg ml$^{-1}$ compared with 112 (72%) of the 155 patients with an aneuploid tumour.

The site of the tumours was not related to an elevated CEA level (Table 2). Interestingly, of the 164 men in the study, only 23 had right-sided tumours compared with 32 out of 113 tumours in women (chi-squared test, $P < 0.005$).

The overall cancer-specific survival for these patients was 52%. If those undergoing palliative surgery (stage D tumours) are excluded, the cancer-specific survival rises to 62%.

The colorectal cancer-specific survival curves for stages A,B,C and D are shown in Figure 1 and, as expected, there is a highly statistically significant difference between the curves. Patients

© Cancer Research Campaign 1998

with a raised preoperative CEA had a worse 5-year survival (39%) than those with a normal preoperative CEA (57%) (Figure 2: hazard ratio 2.04; 95% confidence intervals 1.4–2.9; log rank test $P = 0.0001$). However, when tumour stage was controlled for, the survival benefit of a low preoperative CEA was lost (hazard ratio 1.28; 95% confidence intervals 0.87–1.88; log rank test $P = 0.20$).

### DISCUSSION

Carcinoembryonic antigen (CEA) was first characterized by Gold and Freedman in 1965. It was thought to be produced by carcinomatous or embryonic tissue and not normal adult tissue. However, it soon became apparent that high circulating levels of CEA could be found in non-malignant adult diseases and was capable of being produced by normal adult gastro intestinal secretary cells. Despite this, in 1974 the United States Food and Drug Administration approved marketing of serum CEA levels for the early detection of cancer and screening of patients at high risk of developing colorectal cancer. Since then, the benefit of measuring preoperative serum CEA has been controversial, with different groups reaching different conclusions (Moertel et al, 1986).

Wanebo et al (1978) studied 358 patients with colorectal cancer and found that the recurrence rate was higher in patients with Dukes' B and C lesions if their preoperative CEA was greater than 5 ng ml$^{-1}$, this was highly statistically significant within the two stages. They also found a linear correlation between preoperative CEA levels and estimated mean recurrence time. In contrast to this, Goslin et al, (1980) found no correlation between preoperative CEA levels and risk of tumour recurrence in 71 stage B tumours, but there was a highly significant correlation between CEA levels and recurrence in 46 patients with stage C tumours. Interestingly, if the poorly differentiated stage C tumours are removed, this difference in recurrence rates becomes even more significant. They suggested that poorly differentiated tumours produce little tissue CEA, and this is reflected in low serum CEA levels. However, this study has been unable to confirm this observation; rather an elevated CEA was associated with poorly differentiated tumours.

Lewi et al (1984) obtained similar results to Goslin et al (1980): 102 patients with stage C tumours and a preoperative CEA level greater than 100 μg l$^{-1}$ (equivalent to 40 μg ml$^{-1}$ in Goslin's study) showed a significantly decreased 2- and 5-year survival compared with those with a lower CEA. They found no correlation between preoperative CEA and disease-free survival for stage B tumours.

Moertel et al (1986) measured preoperative CEA in 316 patients undergoing surgery for colorectal cancer and obtained complete 5-year follow-up. Preoperative CEA levels were significantly correlated with stage of disease and with the size of the primary tumour in stage B tumours. There was no association between preoperative CEA and survival for stage A, B or C tumours with 1–3 nodes involved. However, it was an independent predictor of survival in patients whose tumours had four or more metastatically involved lymph nodes. This finding, which is similar to our results, has also been confirmed by Filella et al (1992). Others using a panel of serum markers including CEA measured preoperatively have also been unable to show any benefit over Dukes' staging in predicting survival (Diez et al, 1994; Lindmark et al, 1995).

In contrast, the Japanese report a different experience regarding the ability of preoperative CEA level to predict survival, independent of tumour stage. Multivariate analysis on data pooled from 1254 patients with colorectal cancer revealed that an elevated preoperative CEA as well as nodal involvement and venous invasion were independent factors affecting disease-free survival (Takahashi et al, 1996). Tsuchiya et al (1994), in a series of 124 patients with colorectal cancer, found that the most significant discriminant in prognosis was liver metastasis followed by preoperative CEA level, depth of invasion and DNA ploidy pattern.

In the study reported here, the distribution of tumour stage and cancer-free survival is similar to that reported in other large series. Patients with a raised preoperative CEA have a worse prognosis than those with a normal value. Like many other studies, a relationship between increasing preoperative serum CEA and more advanced tumour stage was found. However, once stage had been controlled for, preoperative CEA gave no useful prognostic information. It is unlikely that a significant effect of a raised CEA on prognosis has been missed, as this study has a power of 80% in detecting a 1.2 difference in the hazard ratios between the groups at the 5% level.

This study also found that a poorly differentiated tumour was associated with a raised preoperative CEA. However, rather surprisingly, there was no relationship between tumour ploidy and CEA level. This finding is in contrast to those of Kouri et al (1991). This may be because serum CEA levels tend to reflect tumour bulk rather than differences in cell production.

This study confirms the findings of other workers in the West that the measurement of preoperative CEA is of little added help in predicting survival once the Dukes' stage is known. Whether this holds true for colorectal cancer in the Far East remains to be seen, and it may be fruitful to explore whether this discrepancy reflects biological differences in the behaviour of colorectal cancer. Occasionally, small early tumours are excised by endoscopic means, such as transanal endoscopic microsurgery (TEMS), and a raised preoperative CEA measurement in this group of patients may indicate advanced local or disseminated disease, allowing treatment to be tailored to the tumour stage. However, preoperative CEA measurement is of no benefit in predicting survival when radical surgical excision of the tumour is planned. Whether its measurement is of any use in the follow-up of patients who have undergone potentially curative colorectal cancer is a contentious issue, but there are no data to indicate that its measurement allows earlier detection of tumour recurrence and increases patient survival (McCall et al, 1994; Vauthey et al, 1996).

This study concludes that a raised preoperative serum CEA is associated with a poorer prognosis and more advanced tumour stage but is of little value in predicting patient survival once the Dukes' stage is known.

### ACKNOWLEDGEMENT

Grateful thanks are given to Ben Palmer for statistical advice.

### REFERENCES

Armitage NC, Ballantyne KC, Sheffield JP, Clarke P, Evans DF and Hardcastle JD (1991) A prospective evaluation of the effect of tumor cell DNA content on recurrence in colorectal cancer. *Cancer* **67**: 2599–2604

Carpelan-Holmstrom M, Haglund C, Lundin J, Jarvinen H and Roberts P (1996) Preoperative serum levels of CA 242 and CEA predict outcome in colorectal cancer. *Eur J Cancer* **32A**: 1156–1161

Chapman MA, Hardcastle JD and Armitage NC (1995) Five-year prospective study of DNA tumor ploidy and colorectal cancer survival. *Cancer* **76**: 383–387

Diez M, Cerdan FJ, Pollan M, Maestro ML, Ortega MD, Martinez S, Moreno G and Balibrea JL (1994) Prognostic significance of preoperative serum CA 19.9 assay in patients with colorectal carcinoma. *Anticancer Res* **14**: 2819–2825

 © Cancer Research Campaign 1998

Dukes CE (1932) The classification of cancer of the rectum. *J Pathol Bacteriol* **35**: 323–332

Eskelinen M. Pasanen P. Kulju A. Janatuinen E. Miettinen P. Poikolainen E. Tarvainen R. Nuutinen P. Paakkonen M and Alhava E (1994) Clinical evaluation of serum tumour markers CEA. CA 50 and CA 242 in colorectal cancer. *Anticancer Res* **14**: 1427–1432

Filella X. Molina R. Grau JJ. Pique JM. Garcia-Valdecasas JC. Astudillo E. Biete A. Bordas JM. Novell A. Campo E and Ballesta AM (1992) Prognostic value of CA19.9 levels in colorectal cancer. *Ann Surg* **216**: 55–59

Filella X. Molina R. Pique JM. Grau JJ. Garcia-Valdecasas. JC. Biete A. Novell F. Astudillo E. Bordas JM. Campo E and Ballesta AM (1994) CEA as a prognostic factor in colorectal cancer. *Anticancer Res* **14**: 705–708

Gold P and Freedman SO (1965) Specific carcinoembryonic antigen of the human digestive system. *J Exp Med* **122**: 467–481

Goslin R. Steele G. Macintyre J. Mayer R. Sugarbaker P. Cleghorn K. Wilson R and Zamcheck N (1980) The use of preoperative plasma CEA levels for the stratification of patients after curative resection of colorectal cancers. *Ann Surg* **192**: 747–751

Kouri M. Pyrhonen S. Mecklin JP. Jarvinen H. Laasonen A. Franssila K. Kuusela P and Nordling S (1991) Serum carcinoembryonic antigen and DNA ploidy in colorectal carcinoma. A prospective study. *Scand J Gastroenterol* **26**: 812–818

Lewi H. Blumgart LH. Carter DC. Gillis CR. Hole D. Ratcliffe JG. Wood CB and McArdle CS (1984) Preoperative carcinoembryonic antigen and survival in patients with colorectal cancer. *Br J Surg* **71**: 206–208

Lindmark G. Bergstrom R. Pahlman L and Glimelius B (1995) The association of preoperative serum tumour markers with Dukes' stage and survival in colorectal cancer. *Br J Cancer* **71**: 1090–1094

McCall JL. Black RB. Rich CA. Harvey JR. Baker RA. Watts JM and Toouli J (1994) The value of serum carcinoembryonic antigen in predicting recurrent disease following curative resection of colorectal cancer. *Dis Colon Rectum* **37**: 875–881

Moertel CG. O'Fallon JR. Go VLW. O'Connell MJ and Thynne GS (1986) The preoperative carcinoembryonic antigen test in the diagnosis. staging and prognosis of colorectal cancer. *Cancer* **58**: 603–610

Northover J (1995) The use of prognostic markers in surgery for colorectal cancer. *Eur J Cancer* **31A**: 1207–1209

Takahashi T. Kato T. Kodaira S. Koyama Y. Sakabe T. Tominaga T. Hamano K. Yasutomi M and Ogawa N (1996) Prognostic factors of colorectal cancer. Results of multivariate analysis of curative resection cases with or without adjuvant chemotherapy. *Am J Clin Oncol* **19**: 408–415

Tsuchiya A. Ando Y. Kikuchi Y. Kanno M. Sato H. Yoshida T and Abe R (1994) Reappraisal of preoperative carcinoembryonic antigen levels as a prognostic factor in resectable colorectal cancer. *Fukushima J Med Sci* **40**: 9–17

Vauthey JN. Dudrick PS. Lind DS and Copeland 3rd EM (1996) Management of recurrent colorectal cancer: another look at carcinoembryonic antigen-detected recurrence. *Dig Dis* **14**: 5–13

Wanebo HJ. Rao B. Pinsky CM. Hoffman RG. Stearns M. Schwartz MK and Oettgen HF (1978) Pre-operative carcinoembryonic antigen level as a prognostic indicator in colorectal cancer. *N Engl J Med* **299**: 448–451

Wang JY. Tang R and Chiang JM. (1994). Value of carcinoembryonic antigen in the management of colorectal cancer. *Dis Colon Rectum* **37**: 272–277

Williams NS. Jass JR and Hardcastle JD (1988) Clinicopathological assessment and staging of colorectal cancer. *Br J Surg* **75**: 649–652

© Cancer Research Campaign 1998